# Sperm Ribosomal DNA Promoter Methylation Levels Are Correlated With Paternal Aging and May Relate With *in vitro* Fertilization Outcomes

**DOI:** 10.3389/fgene.2020.00319

**Published:** 2020-04-03

**Authors:** Lejun Li, Hongping Li, Yonghong Tian, Minhao Hu, Fang Le, Liya Wang, Xiaozhen Liu, Fan Jin

**Affiliations:** ^1^Department of Reproductive Endocrinology, Women’s Hospital, School of Medicine, Zhejiang University School of Medicine, Hangzhou, China; ^2^Key Laboratory of Reproductive Genetics, Ministry of Education, Hangzhou, China

**Keywords:** paternal aging, sperm, rDNA promoter methylation, IVF outcome, fertilization rate

## Abstract

The impact of aging on reproductive outcomes has received considerable critical attention; however, there is much less information available on the effects of paternal age compared to the effects of maternal age. In this study, methylation levels of sperm rDNA promoter regions and Long Interspersed Nucleotide Element 1 (LINE-1) were measured using pyrosequencing and fertilization, day 3 good-quality embryo, pregnancies, and implantation results were assessed. We observed significantly increasing levels of DNA methylation in the sperm rDNA promoter regions with age based on stratifying the samples by age alone (*P* = 0.0001) and performing linear regression analysis (*P* < 0.0001). Meanwhile, no statistically significant correlations were observed between global LINE-1 methylation with age. No statistically significant correlations were observed between sperm rDNA promoter methylation levels and either the day 3 good-quality embryo rate or clinical pregnancy rate. In contrast, the correlation between sperm rDNA promoter methylation levels and fertilization (2 pronuclei) rate was nearly significant (*P* = 0.0707), especially the methylation levels of some individual CpG units (CpG_10, *P* = 0.0176; CpG_11, *P* = 0.0438; CpG_14, *P* = 0.0232) and rDNA promoter methylation levels measured using primerS2 (*P* = 0.0513). No significant correlation was found between sperm rDNA promoter methylation levels and fertilization rates (2 pronuclei, 1 pronuclei, and 1 polypronuclei). Our results demonstrate that sperm are susceptible to age-associated alterations in methylation levels of rDNA promoter regions, suggesting that sperm rDNA promoter methylation levels can be applied to DNA methylation-based age prediction, and that the aberrant methylation of rDNA promoters may be partially responsible for enhanced disease susceptibility of offspring sired by older fathers. Methylation levels of sperm rDNA promoter regions may correlate with polypronuclei rates of IVF programs.

## Introduction

The impact of aging on reproductive outcomes has received considerable critical attention, as there are significant trends toward delayed births. In contrast to the effects of maternal age, there is much less information about the effects of paternal age. Advanced paternal age is associated with delayed time to pregnancy and an increased risk of neuropsychiatric diseases in offspring (Malaspina et al., 2001; Speicher et al., 2010; Nybo Andersen and Urhoj, 2017). Although the causes remain unclear, there is some evidence that alterations in DNA methylation likely occur with increasing age (Jenkins et al., 2015; Kimura et al., 2018). And recent research investigated sperm DNA methylation patterns in 17 fertile donors collected from each individual 9–19 years apart using a methylation array approach and identified 139 regions that are significantly hypomethylated with age and 8 regions that are significantly hypermethylated with age (Jenkins et al., 2014).

The ribosomal RNA (rRNA) genes encode the three major RNAs making up the ribosome that are essential for growth and development (Farley-Barnes et al., 2018). Human pre-implantation development requires the degradation of maternal transcripts and activation of the embryonic genome during the first 3 days after fertilization (Tohonen et al., 2015). rRNA gene activation and associated nucleolus formation can be used as a marker for the activation of the embryonic genome and for evaluation of the developmental potential of embryos (Hyttel et al., 2000). The expression of rRNA genes is subject to complex regulation, including methylation of the ribosomal DNA (rDNA) promoter (Ghoshal et al., 2004; Santoro and Grummt, 2005).

This study aimed to assess the between correlation of sperm rDNA promoter methylation levels and paternal age and evaluate their influence on *in vitro* fertilization (IVF) outcomes. In order to investigate if sperm rDNA promoter methylation levels could be linked to global methylation level of the sperm genome, the methylation status of LINE-1 transposon was then analyzed.

## Materials and Methods

### Patient Selection

This study was conducted among patients enrolled in the assisted reproduction program at Women’s Hospital, School of Medicine, Zhejiang University, Hangzhou, Zhejiang, China from April 2018 to May 2019. All the patients had normal chromosome karyotypes and the male patients had no Y chromosome microdeletions. The exclusion criteria for males were evidence of spermatogenic impairment, including seminal infection, varicocele, and a history of cryptorchidism or orchitis. The Institutional Review Board at Women’s Hospital approved this study. All participants provided written informed consent.

### *In vitro* Fertilization

Semen analysis for sperm count and motility was performed according to protocols from the World Health Organization. Normal fertilization was assessed by the presence of two pronuclei (2PN) and two polar bodies. Polypronuclear fertilization was defined as the presence of three or more pronuclei. The morphological evaluation of all the embryos was performed on day 3 after oocyte retrieval. A good-quality embryo was defined as described previously (Cui et al., 2015): 7–9 cells with equally sized, mononucleated blastomeres and <10% fragmentation. A clinical pregnancy was identified by a positive plasma β-human chorionic gonadotrophin (β-HCG) concentration and the visualization of a fetal heartbeat using ultrasound after 6 weeks. Implantation rates were determined by dividing the number of gestation sacs identified using ultrasound visualization by the number of embryos transferred.

### DNA Isolation, Genotyping and Pyrosequencing

All experiments were performed using motile sperm isolated using density gradients (Isolate, Irvine Scientific) following the manufacturer’s instructions. After the completion of IVF, the remaining spermatozoa were washed in PBS and stored at −80°C until use, as previously described by Goodrich et al. (2007). After thawing, spermatozoa were washed twice in PBS and resuspended in somatic cell lysis buffer (0.1% SDS and 0.5% Triton X-100 in distilled water). The DNA from the spermatozoa was then extracted by a modified guanidinium thiocyanate method (Gazquez et al., 2008). Because of inter-individual variation of rDNA, as well as variation with aging (Malinovskaya et al., 2018), genotyping of rDNA promoter region underwent targeted PCR analysis with Sanger sequencing. PCR primers were: Forward, TTCGGTCCCTCGTGTGTC; Reverse, GAGAGAACAGCAGGCCCG. Bisulfite conversion of each DNA sample was performed using Qiagen EpiTect Bisulfite Kit (Qiagen, United States), according to the manufacturer’s protocol. For bisulfite DNA sequencing, 2 μL of the post-bisulfite-treated product was amplified by PCR. The reaction program included 3 min of heat activation of the enzyme at 95°C followed by 40 cycles of template denaturation at 94°C for 30 s, primer annealing at 56°C for 30 s, and extension at 72°C for 1 min then ending with one cycle at 72°C for 7 min. PCR products were then pyrosequenced using a PyroMark Q96 ID System (Qiagen, United States). PCR primers used to detect LINE-1 methylation level as described previously (Braiteh et al., 2008; Hogg et al., 2014) were: Forward, TTTTGAGTTAGGTGTGGGATATA; Reverse, Bio-AAAATCAAAAAATTCCCTTTC; sequencing primer, AGTTAGGTGTGGGATATAGT. Primer sequences and sequences of rDNA promoter regions whose methylation levels were analyzed are presented in Table 1 and Supplementary Figure S1.

**TABLE 1:** Sequences used in DNA methylation analysis of rDNA promoter by pyrosequencing.

**Parameter**	**DNA sequence (5′ → 3′)**
Forward primer	GTG TGT TTT GGG GTT GAT TAG AG
Reverse primer	5′-biotin-AAA ACC CAA CCT CTC CAA C
Sequencing primer(S1)	GGG TTG ATT AGA GGG T
Sequence to Analyze(S1)	TTY GGG YGT TTY GTG TGT GGT TGY GAT GGT GGY GTT TTT GGG GAT AGG TGT T
Sequencing primer(S2)	TTT TGG GGA TAG GTG
Sequence to analyze(S2)	TTY GTG TYG YGY GTY GTT TGG GTY GGY GGY GTG GTY GGT GAY GYG ATT TTT
Sequencing primer(S3)	GTT TAG GGG GAG GTA TAT T
Sequence to Analyze(S3)	TTT YGT TTY GAG TYG GTA TTT TGG GTY GTY GGG TTA TTG TTG ATA

### Statistical Analysis

ANOVA and Student’s *t*-test were used to compare continuous variables. Pearson’s correlation was calculated in order to analyze the association between methylation level and patient age, semen parameters. *P* < 0.05 was considered significant. All tests were two-sided. Statistical analyses were performed using SPSS software (version 18.0, SPSS Corp., Chicago, IL, United States).

## Results

The present study included 60 couples who underwent IVF (corresponding to 60 cycles, 1 cycle per couple) and embryo transfer. Baseline characteristics of this study are summarized in Table 2. Data are reported as mean ± standard deviation or as median (range).

**TABLE 2:** Patient information (*n* = 60).

**Parameter**	
**Male**	
Age (years)	34.407.20
BMI (kg/m2)	24.023.39
Sperm concentration (million/ml)	64.1216.70
Sperm progressive motility (%)	38.228.41
**Female**	
Age (years)	32.425.77
BMI (kg/m2)	21.902.74
Basal FSH(IU/l)	6.601.99
Basal serum LH (IU/l)	5.622.97
Basal serum E2	124.9 (10-373.8)
Serum T (nmol/l)	0.670.57
Gonadotrophin duration (days)	9.921.87
Gonadotrophin dosage (IU)	2100 (800–3900)
**Fertilization laboratory parameters and clinical pregnancy**	
Oocytes retrieved (n)	9.234.47
Fertilization (2 pronuclei) rate (%)	80.1517.35
Good quality embryo rate (%)	51.2127.90
Embryos transferred(n)	1.700.46
Clinical pregnancy (%)	54.72
Implantation rate (%)	41.11
Miscarriages or losses (n)	5

*BMI, body mass index; FSH, follicle stimulating hormone; LH, luteinizing hormone;E2, estradiol; T, androgen. Data are reported as mean ± standard deviation or as median (range).*

**FIGURE 1:**
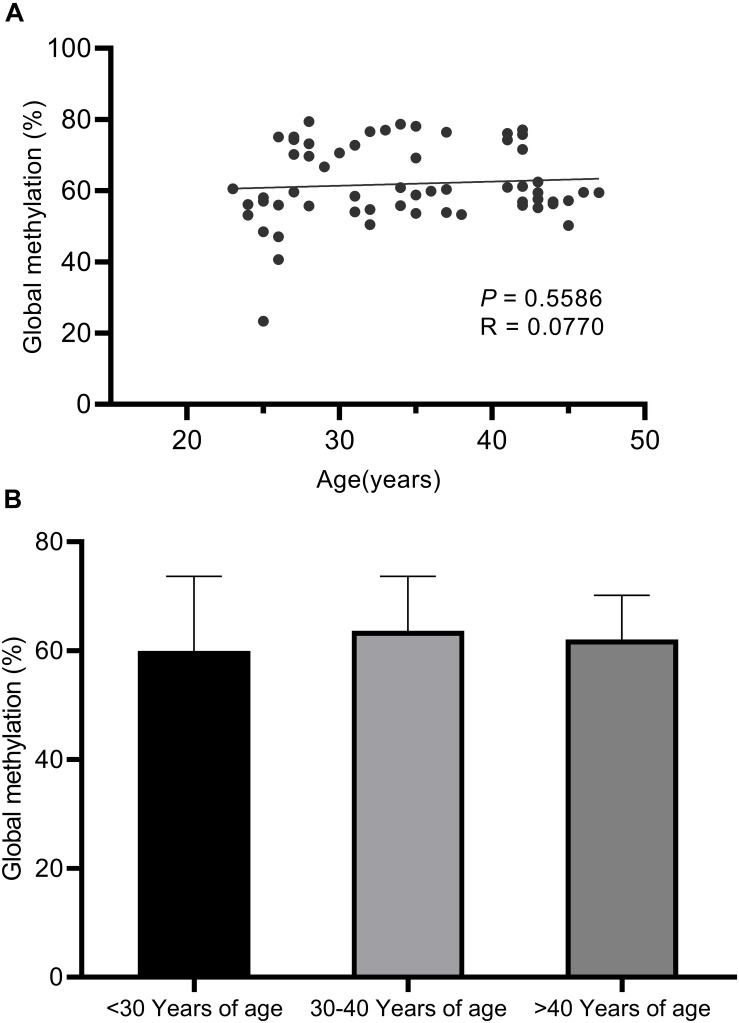
Pyrosequencing results for global LINE-1 methylation assays. **(A)** No statistically significant correlations were observed between global LINE-1 methylation with age based on linear regression analysis (*P* < 0.5586). **(B)** No statistically significant correlations were observed between global LINE-1 methylation with age based on ANOVA analysis (*P* = 0.5602) and unpaired *t*-tests between the three age groups (<30 years of age, 30–40 years of age, >40 years of age).

Long Interspersed Nucleotide Element 1 transposon, which comprise approximately 17% of the human genome, was used as a surrogate marker for global methylation (Yang et al., 2004). No statistically significant correlations were observed between global LINE-1 methylation with age (Figure 1).

Twenty- one CpG residues in sperm rDNA promoters regions were assayed using 3 different primer sets (Table 1 and Supplementary Figure S1). The mean mCpG density ± SD in the sperm rDNA promoter of all the male patients was 16.93 ± 4.05%. The sequences examined by pyrosequencing were identical for all analyzed samples and were identical to the published rDNA gene sequence U13369.

**FIGURE 2:**
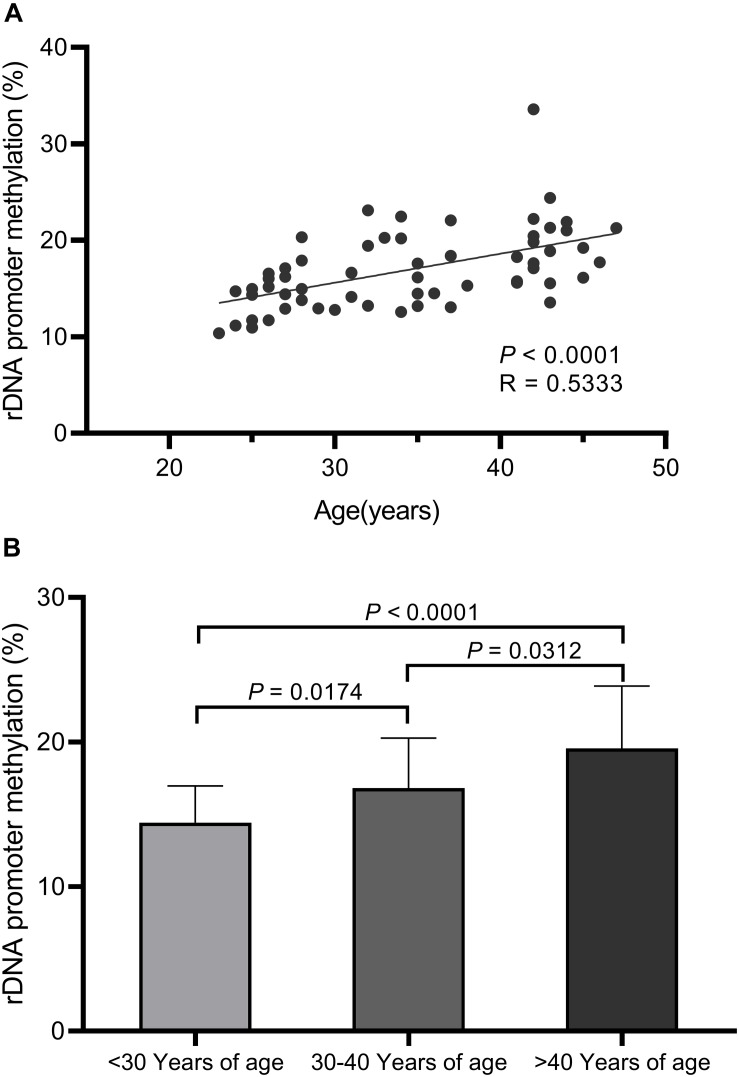
Pyrosequencing results for sperm rDNA promoter methylation assays. **(A)** Linear regression analysis confirms the significant increases in sperm rDNA promoter methylation levels with age (*P* < 0.0001). **(B)** Sperm rDNA promoter methylation levels significantly increase with age based on ANOVA analysis (*P* = 0.0001) and unpaired *t*-tests between the three age groups (<30 years of age, 30–40 years of age, >40 years of age).

We observed significant increases in DNA hypermethylation in the rDNA promoter region of sperm DNA with age (Figure 2) by stratifying samples only by age (*P* = 0.0001) and performing linear regression analysis (*P* < 0.0001). When all three age groups (<30 years, 30–40 years, >40 years of age) were compared, a significant correlation between DNA hypermethylation levels in sperm rDNA promoter regions with age was observed. We also analyzed rDNA promoter methylation using multiple pyrosequencing primers (Supplementary Figures S2–S4) and found that DNA methylation significantly increases with age, especially when comparisons were performed using primer S2 (Supplementary Figure S3). No statistically significant correlation was observed between rDNA promoter methylation and either sperm concentration or progressive motility (data not shown). Embryonic developmental effects were assessed based on levels of fertilization (2 pronuclei), day-3 good-quality embryos, and clinical pregnancies. No statistically significant correlation was found between sperm rDNA promoter methylation levels and either day-3 good-quality embryo rates or clinical pregnancy rates (Tables 3, 4). However, the correlation between sperm rDNA promoter methylation levels and fertilization (2 pronuclei) rate was nearly significant (*P* = 0.0707), especially the methylation levels of some individual CpG units (CpG_10, *P* = 0.0176; CpG_11, *P* = 0.0438; CpG_14, *P* = 0.0232) and rDNA promoter methylation levels measured using primerS2 (*P* = 0.0513) (Table 3). When the fertilization rate was categorized into two categories (<70% and ≥70%), rDNA promoter methylation levels were higher in the ≥70% fertilization (2 pronuclei) rate group (*P* = 0.093) (Table 4), especially when rDNA promoter methylation levels were measured with primer S2 (*P* = 0.072, data not shown). However, no statistically significant correlation was found between sperm rDNA promoter methylation levels and fertilization rates (2 pronuclei, 1 pronuclei, and 1 polypronuclei).

**TABLE 3:** Regression of Fertilization (2 pronuclei) rate and good-quality embryo rate on rDNA promoter methylation level (mean values or individual CpG unit).

**rDNA promoter methylation level (mean values or CpG unit)**	**Fertilization (2 pronuclei) rate**	**Good-quality embryo rate on day 3**
Mean methylation values of all CpG units	0.2350 (*P* = 0.0707)	0.0954 (*P* = 0.4684)
Mean methylation values measured using primerS1	0.2070 (*P* = 0.1126)	0.0754 (*P* = 0.5670)
Mean methylation values measured using primerS2	0.2528 (*P* = 0.0513)	0.1002 (*P* = 0.4461)
Mean methylation values measured using primerS3	0.1857 (*P* = 0.1555)	0.0874 (*P* = 0.5068)
CpG_1	0.2274 (*P* = 0.0806)	0.0446 (*P* = 0.7354)
CpG_2	0.0336 (*P* = 0.7987)	0.0988 (*P* = 0.4526)
CpG_3	0.2371 (*P* = 0.0682)	0.0649 (*P* = 0.6223)
CpG_4	0.2316 (*P* = 0.0749)	0.0867 (*P* = 0.5100)
CpG_5	0.1108 (*P* = 0.3995)	0.0852 (*P* = 0.5176)
CpG_6	0.2419 (*P* = 0.0626)	0.1042 (*P* = 0.4283)
CpG_7	0.2512 (*P* = 0.0529)	0.1354 (*P* = 0.3022)
CpG_8	0.2459 (*P* = 0.0582)	0.0763 (*P* = 0.5623)
CpG_9	0.2010 (*P* = 0.1235)	0.1026 (*P* = 0.4354)
CpG_10	0.3055 (*P* = 0.0176)*	0.1383 (*P* = 0.2920)
CpG_11	0.2613 (*P* = 0.0438)*	0.0475 (*P* = 0.7185)
CpG_12	0.2049 (*P* = 0.1163)	0.1024 (*P* = 0.4360)
CpG_13	0.2032 (*P* = 0.1194)	0.0790 (*P* = 0.5484)
CpG_14	0.2928 (*P* = 0.0232)*	0.1041 (*P* = 0.4287)
CpG_15	0.1988 (*P* = 0.1278)	0.0945 (*P* = 0.4727)
CpG_16	0.1412 (*P* = 0.2820)	0.0450 (*P* = 0.7329)
CpG_17	0.1866 (*P* = 0.1534)	0.0894 (*P* = 0.4971)
CpG_18	0.1515 (*P* = 0.2479)	0.0854 (*P* = 0.5166)
CpG_19	0.1891 (*P* = 0.1480)	0.0738 (*P* = 0.5750)
CpG_20	0.2147 (*P* = 0.0995)	0.0947 (*P* = 0.4716)
CpG_21	0.1951 (*P* = 0.1352)	0.0848 (*P* = 0.5194)
		

*P < 0.05.

**TABLE 4:** The relationship between the primary parameters of embryonic development and rDNA promoter methylation levels.

		**rDNA promoter**	
		**methylation**	
**Primary parameters**	***n***	**levels**	***P* value^#^**
**Fertilization (2 pronuclei) rate**			
≥70%	44	17.68 ± 4.17	0.093
<70%	16	15.68 ± 3.53	
**Good-quality embryo rate on day 3**			
≥60%	25	17.43 ± 3.77	0.649
<60%	35	16.94 ± 4.32	
**Pregnancy (Fresh embryo transfer, n)**			
Yes	11	19.04 ± 6.22	0.169
No	12	16.02 ± 3.76	
**Pregnancy (Frozen embryo transfer, n)**			
YES	18	16.75 ± 3.41	0.865
No	12	16.53 ± 3.50	

*^#^*t*-test.*

## Discussion

This study demonstrates that DNA methylation levels in sperm rDNA promoter regions are maintained at low levels in human sperm, which is expected since DNA methylation is dynamically reprogrammed in primordial germ cells via DNA demethylation (Furuta and Nakamura, 2017). However, repeat elements such as LINE1 transposon are heavily methylated in sperm (Molaro et al., 2011). This study also demonstrates that sperm are susceptible to age-associated alterations of DNA methylation in the rDNA promoter region. During aging, rDNA may undergo *de novo* methylation because of the repetitive nature of rDNA cistrons (Oakes et al., 2003). In addition, the contributions of DNA methylation marks in sperm to embryo development have been identified (Arpanahi et al., 2009; Hammoud et al., 2009; Kropp et al., 2017) and genome-wide sperm DNA methylation can be used to predict male fertility status and embryo quality (Aston et al., 2015). Recent research indicates that some specific sperm genomic regions are especially susceptible to age-associated alterations in DNA methylation, especially those containing genes associated with schizophrenia and bipolar disorder (Jenkins et al., 2014). These data indicate that the sperm methylation may also contribute to events beyond fertilization, like pre-implantation development.

Embryonic genome activation (EGA) is probably most important during the pre-implantation period of mammalian development, in which transcripts expressed from the embryonic genome replace the maternal transcripts (Kanka, 2003). Human EGA most likely occurs during the first 3 days after fertilization (Tohonen et al., 2015). rRNA gene activation and associated nucleolus formation can be used as markers for EGA and in evaluating the developmental potential of embryos (Hyttel et al., 2000). rRNA genes encode the three major RNAs composing the ribosome and are thus essential for growth and development. The expression of rRNA genes is subject to complex regulation, including the methylation of rDNA promoter regions (Ghoshal et al., 2004; Santoro and Grummt, 2005). Several studies demonstrate that the levels of rDNA promoter region methylation are correlated with rRNA expression (Stancheva et al., 1997; Santoro and Grummt, 2001). In this study, we failed to detect an association between sperm rDNA promoter methylation status and embryonic developmental effects. Previous studies have shown aberrant global sperm DNA methylation impacts male fertility status and embryo quality (Benchaib et al., 2005; Aston et al., 2015). The difference between results from previous studies and our work is likely explained by the fact that all experiments in our study were performed using motile sperm and excluding immature or incompletely compacted sperm and by differences in methods used to detect DNA methylation. In addition, the correlation between sperm rDNA promoter methylation levels and fertilization (2 pronuclei) rate nearly reached significance, and no significant correlation was found between sperm rDNA promoter methylation levels and the fertilization rate (2 pronuclei, 1 pronuclei, and 1 polypronuclei). This suggests that abnormal fertilization (1 pronuclei and 1 polypronuclei) occurs in patients with lower methylation levels in sperm rDNA promoter regions. Because polypronuclei fertilization, a good predictor of pregnancy outcomes (Yie et al., 1996; Shibahara et al., 2003), mainly occurs during conventional IVF (Gardner and Simón, 2017), it may provide clues about the relationship between aberrant sperm rDNA promoter methylation and embryonic developmental effects. To strengthen this conclusion, a larger-scaled study is needed to validate our results.

## Conclusion

In conclusion, we found that rDNA promoter hypermethylation in human sperm was associated with paternal aging, suggesting that sperm rDNA promoter methylation levels can be applied to DNA methylation-based age prediction, and considering the importance of ribosome for growth and development, aberrant methylation of rDNA promoters may be partly responsible for levels of disease susceptibility of offspring sired by older fathers.

## Data Availability Statement

The datasets generated for this study are available on request to the corresponding author.

## Ethics Statement

The studies involving human participants were reviewed and approved by The Institutional Review Board at Women’s Hospital, School of Medicine, Zhejiang University, Hangzhou, Zhejiang, China. The patients/participants provided their written informed consent to participate in this study.

## Author Contributions

FJ and LL project administration and conceptualization. HL, MH, and FL sampling and DNA extraction. LW and XL pyrosequencing. LL and YT data analysis and writing. FJ and LL article review. All authors discussed results and commented on the manuscript.

## Conflict of Interest

The authors declare that the research was conducted in the absence of any commercial or financial relationships that could be construed as a potential conflict of interest.

## Supplementary Material

FIGURE S1 The human rDNA promoter region. **(A)** PCR primer sequences for DNA methylation analysis of rDNA promoter are indicated by the underlining. The sequences examined by pyrosequencing are boxed, with the location marked based on its position relative to the transcriptional start site (arrow) and CpG dinucleotides marked in red. **(B)** Chromatograms corresponding to the sequences examined by pyrosequencing PrimerS1. **(C)** Chromatograms corresponding to the sequences examined by pyrosequencing PrimerS2. **(D)** Chromatograms corresponding to the sequences examined by pyrosequencing PrimerS3. The sequences of the rDNA promoter as shown were identical for all analyzed.

FIGURE S2 Pyrosequencing results for sperm rDNA promoter methylation assays with primer S1. **(A)** Linear regression analysis confirms the significant increases in sperm rDNA promoter methylation levels with age (*P* = 0.0008). **(B)** Sperm rDNA promoter methylation levels significantly increase with age based on ANOVA analysis (*P* = 0.0033) and unpaired *t*-tests between the <30-year-old age group and the 30-40-year-old age group and between the <30-year-old age and the >40-year-old age group. However, no significantly increase with age was observed between the 30–40-year-old age group and the >40-year-old age group using the unpaired *t*-test.

FIGURE S3 Pyrosequencing results for sperm rDNA promoter methylation assays with primer S2. **(A)** Linear regression analysis confirms the significant increases in sperm rDNA promoter methylation levels with age (*P* < 0.0001). **(B)** Sperm rDNA promoter methylation levels significantly increase with age based on ANOVA analysis (*P* < 0.0001) and unpaired *t*-tests between the three age groups (<30 years of age, 30–40 years of age, and >40 years of age).

FIGURE S4 Pyrosequencing results for sperm rDNA promoter methylation assays with primer S3. **(A)** Linear regression analysis confirms the significant increase in sperm rDNA promoter methylation levels with age (*P* = 0.0127). **(B)** Sperm rDNA promoter methylation levels significantly increase with age based on unpaired *t*-test between the <30-year-old age group and the >40-year-old age group, but did not significantly increase with age based on ANOVA analysis (*P* = 0.0562) and unpaired *t*-tests between the <30-year-old age group and the 30-40-year-old age group or between the 30–40-year-old age group and the >40-year-old age group.
