# Supplementary figures and images for: Sperm Ribosomal DNA Promoter Methylation Levels Are Correlated With Paternal Aging and May Relate With in vitro Fertilization Outcomes
Source: Front Genet. 2020 Apr 3;11:319. doi: 10.3389/fgene.2020.00319 (PMC7147477; doi:10.3389/fgene.2020.00319)

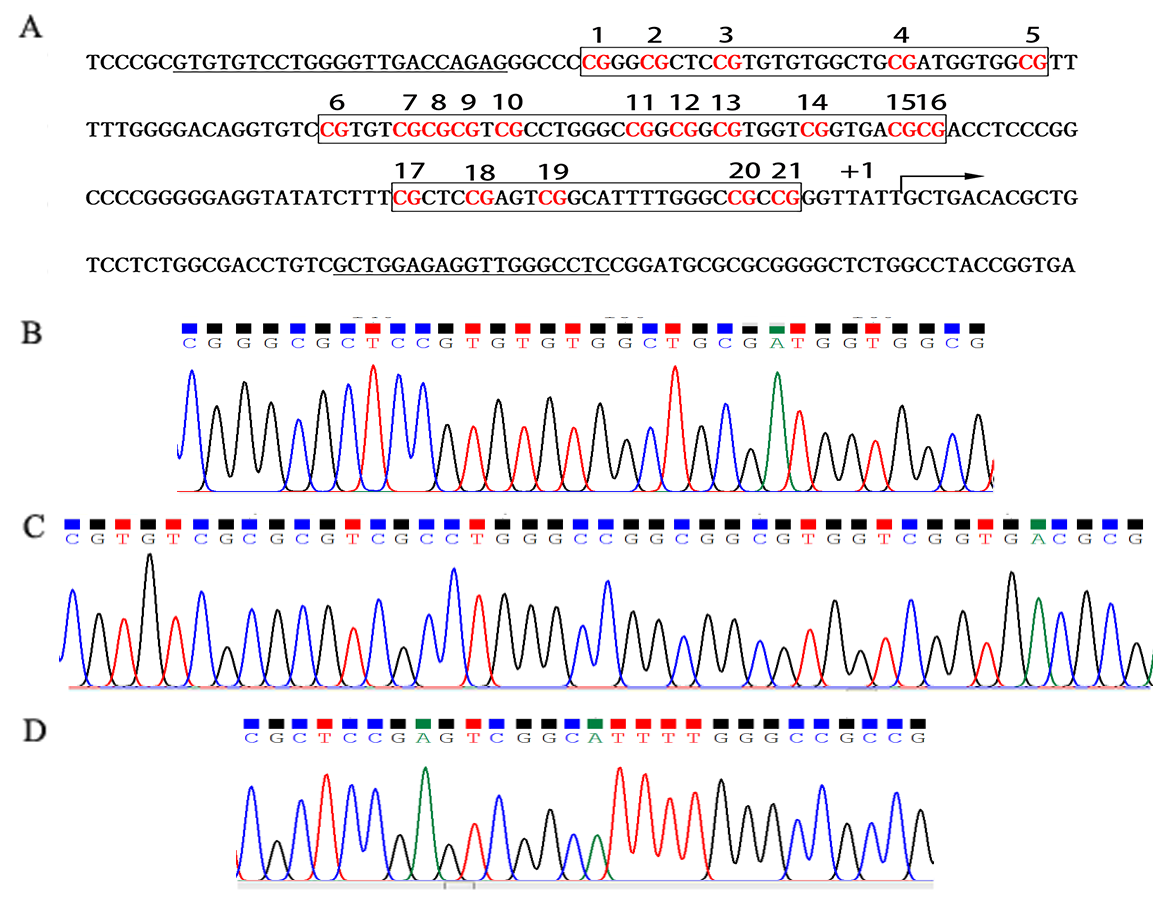

Supplement: FIGURE S1 — The human rDNA promoter region. (A) PCR primer sequences for DNA methylation analysis of rDNA promoter are indicated by the underlining. The sequences examined by pyrosequencing are boxed, with the location marked based on its position relative to the transcriptional start site (arrow) and CpG dinucleotides marked in red. (B) Chromatograms corresponding to the sequences examined by pyrosequencing PrimerS1. (C) Chromatograms corresponding to the sequences examined by pyrosequencing PrimerS2. (D) Chromatograms corresponding to the sequences examined by pyrosequencing PrimerS3. The sequences of the rDNA promoter as shown were identical for all analyzed. [file Image_1.TIF]

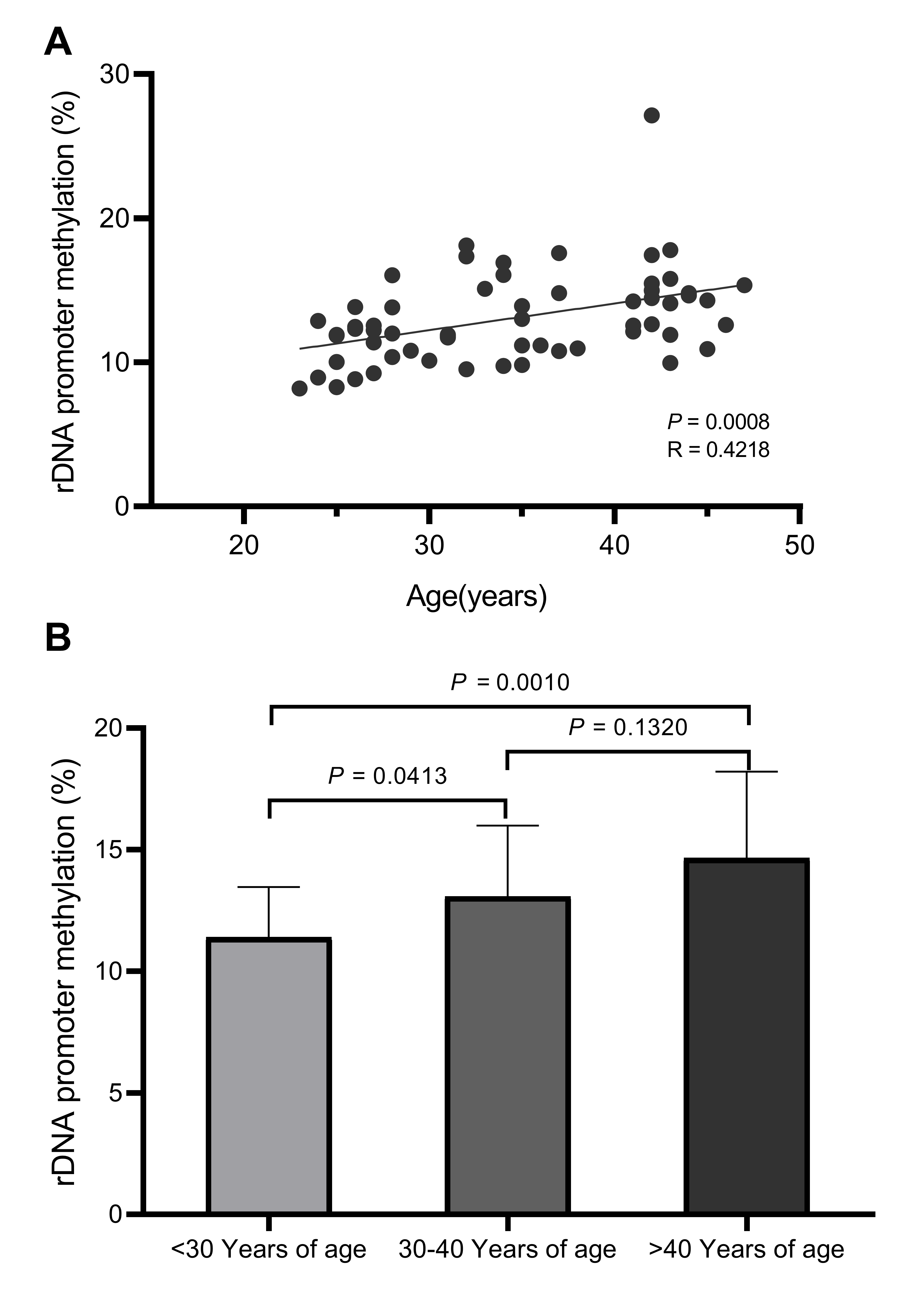

Supplement: FIGURE S2 — Pyrosequencing results for sperm rDNA promoter methylation assays with primer S1. (A) Linear regression analysis confirms the significant increases in sperm rDNA promoter methylation levels with age (P = 0.0008). (B) Sperm rDNA promoter methylation levels significantly increase with age based on ANOVA analysis (P = 0.0033) and unpaired t-tests between the <30-year-old age group and the 30-40-year-old age group and between the <30-year-old age and the >40-year-old age group. However, no significantly increase with age was observed between the 30–40-year-old age group and the >40-year-old age group using the unpaired t-test. [file Image_2.TIF]

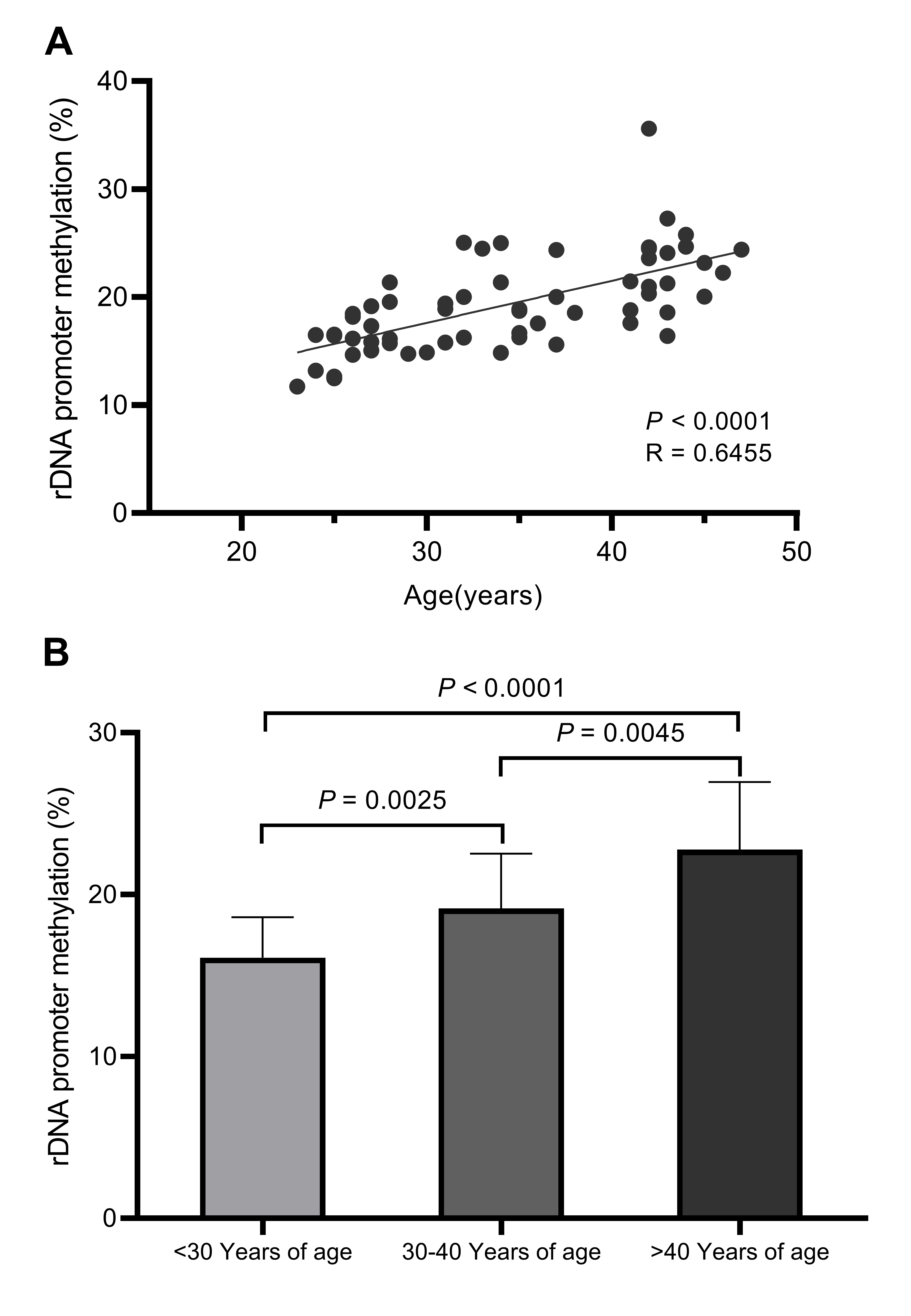

Supplement: FIGURE S3 — Pyrosequencing results for sperm rDNA promoter methylation assays with primer S2. (A) Linear regression analysis confirms the significant increases in sperm rDNA promoter methylation levels with age (P < 0.0001). (B) Sperm rDNA promoter methylation levels significantly increase with age based on ANOVA analysis (P < 0.0001) and unpaired t-tests between the three age groups (<30 years of age, 30–40 years of age, and >40 years of age). [file Image_3.TIF]

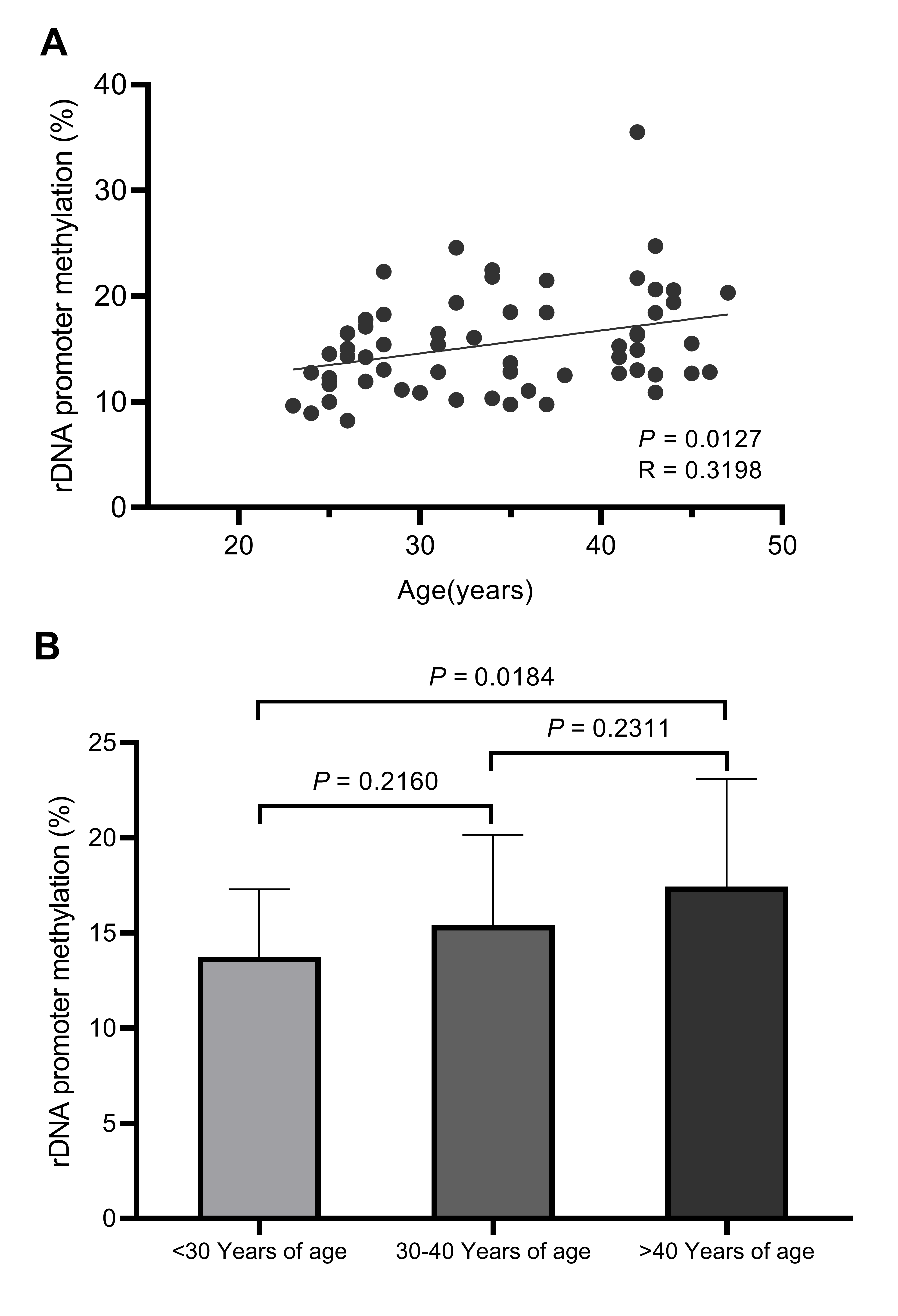

Supplement: FIGURE S4 — Pyrosequencing results for sperm rDNA promoter methylation assays with primer S3. (A) Linear regression analysis confirms the significant increase in sperm rDNA promoter methylation levels with age (P = 0.0127). (B) Sperm rDNA promoter methylation levels significantly increase with age based on unpaired t-test between the <30-year-old age group and the >40-year-old age group, but did not significantly increase with age based on ANOVA analysis (P = 0.0562) and unpaired t-tests between the <30-year-old age group and the 30-40-year-old age group or between the 30–40-year-old age group and the >40-year-old age group. [file Image_4.TIF]
